# 57-year-old Female with Unusual Left-arm Movements

**DOI:** 10.5811/cpcem.2022.2.55676

**Published:** 2022-02-28

**Authors:** Christina M. Powell, Lauren S. Rosenblatt, Laura J. Bontempo, Zachary D.W. Dezman

**Affiliations:** * University of Maryland Medical Center, Baltimore, Maryland; † University of Maryland School of Medicine, Department of Emergency Medicine, Baltimore, Maryland; ‡ University of Maryland, Baltimore, Department of Epidemiology and Public Health, Baltimore, Maryland

**Keywords:** stroke, alien hand syndrome, CPC

## Abstract

**Introduction:**

A 57-year-old, right-hand dominant female presented to the emergency department striking herself with her left hand.

**Case Presentation:**

The astute medical staff looked beyond a behavioral health etiology. A detailed history, physical examination, and workup reveals the fascinating final diagnosis.

**Discussion:**

This case takes the reader through the differential diagnosis and systematic workup of uncontrolled limb movements with discussion of the studies which ultimately led to this patient’s diagnosis.

## CASE PRESENTATION (DR. POWELL)

A 57-year-old, right-hand dominant female presented to the emergency department (ED) with unusual movements and shaking of her left arm. In the ED, the staff had to restrain the patient from actively hitting herself in an excited manner. The nursing staff then requested medical evaluation and clearance from the physician team so the patient could be transferred to the psychiatric ED due to apparent self-destructive behavior.

The patient stated she had not been sleeping well recently. Her husband was away and she felt overwhelmed by caring for their dog. The patient stated she awoke that morning around 2:30 am feeling weak to the point that she could not get out of bed. She felt as if her left arm did not belong to her and she could not control her arm’s movements. She had to hold her left hand with her right to keep her left arm from moving. She denied ever having this problem before. Her husband stated that she had previously exhibited similar symptoms when faced with stressors. The patient also complained of numbness and tingling of her left hand and mild shortness of breath. She stated it felt like “cold water is running down my [left] arm.” Additionally, she complained of a feeling in her throat that was making it “hard to breathe,” and she had a headache located in the back of her head, described as slightly worse than her prior headaches. The patient had previously been involuntarily admitted to a behavioral health unit after attempting to harm herself, although she did not have any psychiatric diagnoses.

She had a past medical history of breast cancer status post right mastectomy. She did not take any daily medications and had no drug allergies. Her family history was notable for cancer and coronary artery disease. She was a daily tobacco smoker and occasionally used marijuana. She denied any alcohol use. She denied any chest pain, nausea, vomiting, change in vision, lightheadedness, dropping of objects, or trauma. She recently had a mild, pruritic skin rash for which she completed a course of oral prednisone. She denied joint pain, fevers, chills, cough, or abdominal complaints.

On examination, she was alert and, although she appeared very anxious, was in no acute distress. She was afebrile (37.1° Celsius) with a heart rate of 94 beats per minute, blood pressure 108/62 millimeters of mercury, and her oxygen saturation was 97% while breathing room air. She weighed 51.8 kilograms (kg) (114 pounds), was 165.1 centimeters in height (5 foot 4 inches), and had a body mass index of 20.14 kg per meter^2^. She was well developed and well nourished. Her head was normocephalic and atraumatic. She had dry mucus membranes with a clear oropharynx. Pupils were equal, round, and reactive to light and accommodation with normal extraocular movements and anicteric sclera. Her neck was supple, with full active range of motion, and without lymphadenopathy or carotid bruits. Her lungs were clear to auscultation bilaterally, without wheezes, crackles, or rhonchi. She did not have any increased work of breathing. Her heartbeat was regular at a normal rate without murmurs, rubs, or gallops. The patient’s abdomen was soft with normal bowel sounds and without distention, tenderness, rebound, or guarding. Her extremities had no edema, had 2+ pulses, and were without tenderness or deformity bilaterally. Erythematous scaly plaques were observed on her neck, bilateral ankles, axilla, and lower arms.

Neurologic examination showed cranial nerves II–XII were intact except for a left homonymous inferior quadrantanopia. She had 5/5 strength throughout all extremities and normal muscle bulk and tone. The upper extremity exam was limited by flailing movements of her left arm, which were notably high in amplitude and non-rhythmic. The movements worsened with emotional distress and decreased with distraction. When the patient was asked to look at something specific, the flailing left-arm movements stopped. There was decreased sensation to light touch and pinprick over the left upper extremity in a patchy distribution. Her biceps, brachioradialis, and patellar reflexes were 1+ bilaterally. No dysmetria was noted on finger-nose-finger testing bilaterally, and the patient declined a gait exam. Her speech was clear. She followed commands and answered most questions appropriately but occasionally was tangential. She was oriented to self, place, and time.

**Image 1:**
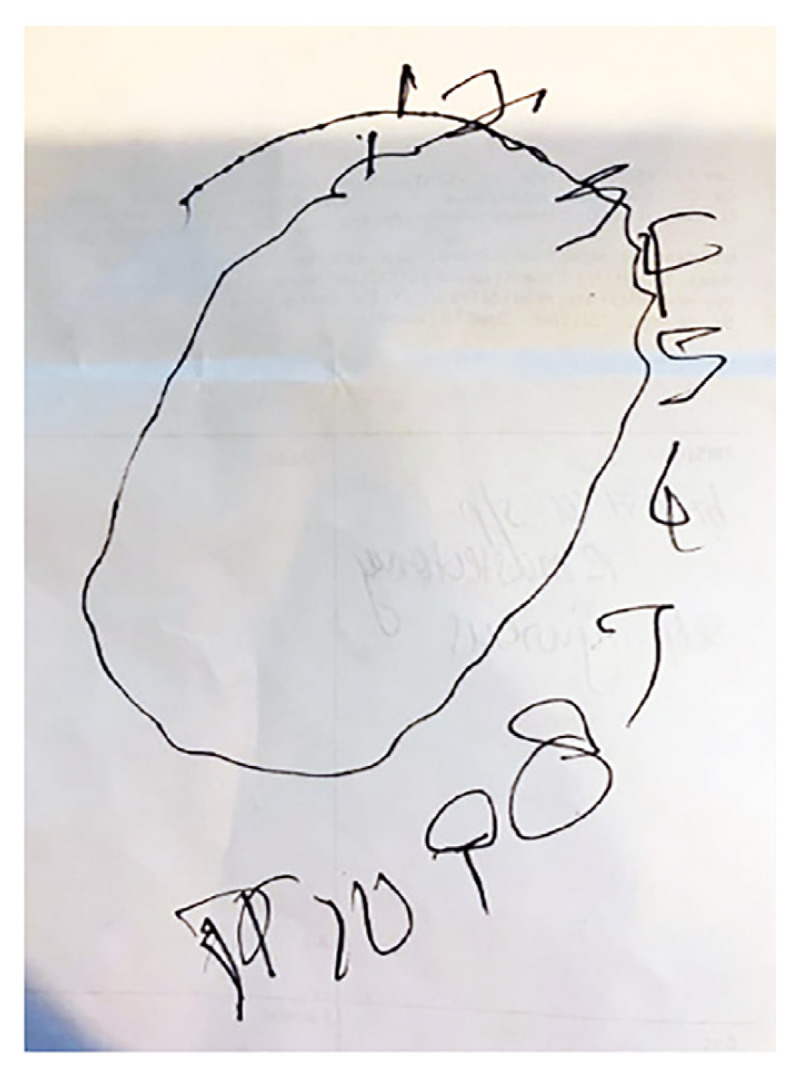
Clock drawn with the dominant (right) hand of a 57-year-old female presenting with strange movements and shaking of her left arm.

Initial laboratory results are shown in Tables 1–2. An electrocardiogram (ECG) was completed, which showed sinus tachycardia without signs of ischemia. A chest radiograph did not show any focal consolidations, pneumothorax, or cardiomegaly. Computed tomography (CT) of the head without contrast did not show evidence of an acute intracranial bleed or acute infarction. There was non-specific asymmetric diffuse sulcal effacement of the right cerebral hemisphere relative to the left. No midline shift was noted. Neurology was consulted due to worsening left upper-extremity movements throughout her ED evaluation. A clock drawing completed by the patient is shown in Image 1. A diagnostic test was then performed, which confirmed the diagnosis.

## CASE DISCUSSION (DR. ROSENBLATT)

This is a case of a 57-year-old female who arrived in the ED with a very unusual presentation that could have easily been dismissed as a mental health issue. This patient is a great reminder that medical etiologies always need to be considered and ruled out alongside psychiatric etiologies. The medical team taking care of this patient astutely noted the unusual presentation and did an extensive exam and workup to consider, and presumably ultimately identify, a treatable medical condition. Now I am tasked with sorting through this presentation and workup to determine what facts are valuable in leading me to a correct final diagnosis.

To summarize, this patient presented to the ED with some striking exam findings. It would be hard not to notice a patient who appeared visibly anxious and was hitting herself repeatedly with her extremity. From the history provided, the patient developed the symptoms of uncontrolled arm movements, paresthesias (described as the sensation of water on her left upper extremity), and a feeling of lack of ownership of the left upper extremity acutely. It was noted that at 2:30 am she felt tired and weak and, upon waking later that morning, developed the unusual movements. Although her husband reports suicidal ideation in the past with some self-injurious behavior of hitting her head against a counter, she does not have any formal psychiatric diagnoses nor any other chronic medical diagnoses in her past medical history.

While the patient’s history certainly is informative, focusing on the physical exam provided the best starting point for developing a working differential diagnosis. The neurologic exam provided the most useful information. There were three exam findings that really stood out: 1) large amplitude flailing left upper extremity movement; 2) left homonymous inferior quadrantanopia; and 3) hemineglect. The left upper extremity movements are noted to be involuntary, large amplitude, and non-rhythmic, which is consistent with a description of chorea and, more specifically, ballismus. The hemineglect is particularly interesting as it is noted in both the history, by the patient expressing lack of ownership of her arm, as well as the physical exam, as demonstrated by her clock drawing (Image 1). It would have been helpful to see whether the patient could draw something from memory, as this would help differentiate visuospatial dysfunction from hemineglect. Additionally, it is important to note other exam findings such as the sensory deficits to light touch and pinprick in the left arm and a rash of unknown chronicity.

Using this information, I started to home in on a differential diagnosis. Just as you would in any clinical setting, it is important to start broadly. Each of the key neurologic findings noted above can be associated with a wide variety of etiologies. These etiologies can then be grouped into autoimmune, endocrine, hereditary, infectious, malignant, metabolic, toxic/drug-induced, traumatic, and vascular categories. Keeping the ballismus, vision changes, and hemineglect in mind, I developed the following list:

- Autoimmune: Sydenham’s chorea; lupus; vasculitis
- Endocrine: Hyperthyroid; hyperparathyroid; hypoparathyroid
- Hereditary: Huntington’s disease; Wilson’s disease; ataxia-telangiectasia; Lesch-Nyhan syndrome
- Infectious: Human immunodeficiency virus (HIV); toxoplasmosis; meningitis; encephalitis
- Malignant: Primary neoplasm; metastatic disease
- Metabolic: Non-ketotic hyperglycemia; hypernatremia; hyponatremia; hypercalcemia; hypocalcemia
- Toxic/Drug induced: Toxic ingestion; side effect of chronic medication use (ie, levodopa, anti-psychotics)
- Traumatic: Head injury
- Vascular: Stroke.

Some of these diagnoses can be ruled out easily. Head trauma can be excluded immediately, as there is no history of recent trauma or head injury in her presentation. The hereditary etiologies of Huntington’s disease, Wilson’s disease, ataxia-telangiectasia, and Lesch-Nyhan are all unlikely diagnoses. All these diseases can be associated with chorea/ballismus but are unlikely to cause other neurologic symptoms. Additionally, many of them would present early in childhood (such as Lesch-Nyhan) or be associated with a strong family history (such as Huntington’s disease). Given the patient’s age and lack of reported family history of neurological problems, these are excluded. The metabolic etiologies can also be ruled out by the normal lab values provided.

Endocrine etiologies were also considered as they can cause neurologic and psychiatric manifestations; however, I would consider neurological and psychiatric symptoms to be the extreme presentation of these diseases and a part of a progression of symptoms. It would be unlikely to have an acute onset of symptoms associated with either hyperthyroidism, hyperparathyroidism, or hypoparathyroidism. When the thyroid is involved, one would expect additional symptoms of thyrotoxicosis such as tachycardia, weight loss, and even exophthalmos. However, this patient does not have any of those additional symptoms. Parathyroid disease can also be ruled out by the normal calcium and phosphorus lab values. Toxic ingestions and drug-induced causes can similarly be excluded. This patient is not currently on any medications and, although she has had a prior presentation for suicidal thoughts, she does not express recent or current thoughts of suicide. Her urine drug screen was positive for marijuana and opiates; however, her constellation of neurologic findings is not consistent with a typical opiate or cannabinoid toxidrome.

Next, I considered autoimmune causes, such as Sydenham’s chorea, lupus, and vasculitis. Sydenham’s chorea is a rare neurologic disorder, characterized by ballismus. It is usually a result of streptococcal infection and rheumatic fever. This is almost always a disorder in children, although there have been extremely rare case reports in adults. The rarity of Sydenham’s chorea would make it an unlikely cause of this patient’s presentation, especially in the absence of recent streptococcal infection or infectious symptoms. Lupus and vasculitis should be considered, as these diseases can affect any part of the body. Isolated acute neurologic presentations are rare, however, as the initial presentations of these diseases. Usually there is progression of symptoms over time. The rash that the patient has can certainly make these etiologies more favorable; however, the chronicity of the rash is unknown to me. Regardless of the duration of the rash, the acuity of this patient’s clinical presentation, without additional history or a review of systems that paints a picture of progression of disease over time, makes an autoimmune diagnosis less likely.

Infection must also be considered. I have identified HIV, toxoplasmosis, meningitis, and encephalitis as possible etiologies. However, the patient does not have a history of HIV or being immunocompromised, making HIV and toxoplasmosis unlikely. Meningitis and encephalitis are still possible, but lower on the differential diagnosis. The patient has not had any recent fevers and was afebrile on presentation. Additionally, she has had no recent complaints of nausea, vomiting, fatigue, decreased appetite, or malaise leading up to her ED presentation. On exam the patient did not have clinical signs of meningismus. The unusual neurologic physical exam findings could be due to central nervous system infection but would be a very uncommon presentation of meningitis or encephalitis.

This leaves me with two unexplored categories: malignancy and vascular. Malignancy is a feasible diagnosis for the patient’s symptoms; however, I would expect a progression of symptoms. The patient does report a headache, but this seems to be a new symptom. A more likely presentation of malignancy would include progressive worsening of her headache, or intermittent or progressive worsening of neurologic symptoms. Additionally, this patient did not report any recent weight loss, night sweats, or fevers in her history. The acuity of the patient’s presentation makes malignancy lower on my differential diagnosis.

Finally, a vascular etiology must be investigated. Remember, this patient presented with the acute onset of ballismus, hemineglect, and inferior quadrantanopia. When revisiting the patient’s neurologic findings, I attempted to localize the areas of the brain that would most commonly need to be affected to cause these symptoms. The symptoms map to the following parts of the brain:

- **Ballismus/chorea** – Caudate, putamen, thalamus, subthalamic nucleus
- **Hemineglect** – Non-dominant parietal lobe infarct (most commonly right side)
- **Inferior quadrantanopia** – Superior optic radiations in the parietal lobe.

All the areas of the brain noted above are primarily supplied by the middle cerebral artery (MCA). Therefore, my final diagnosis is MCA stoke, and the diagnostic test would be magnetic resonance imaging (MRI).

## CASE OUTCOME (DR. POWELL)

**Image 2:**
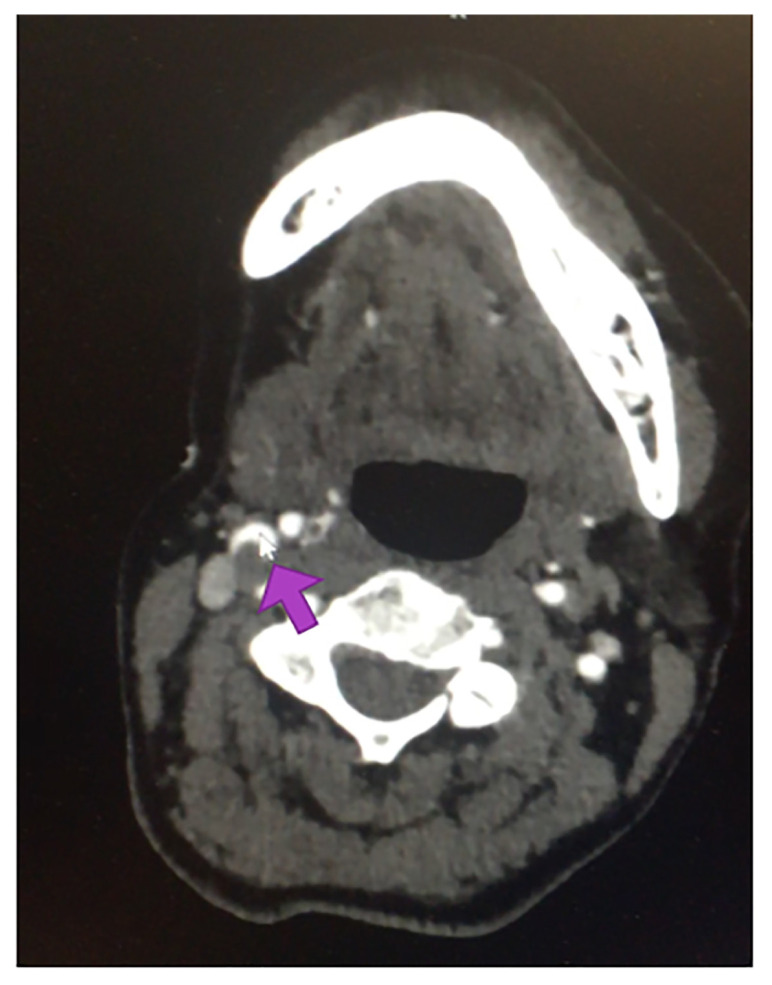
Computed tomography angiography of the neck identifying soft tissue at the right carotid bulb (arrow), mildly narrowing the lumen by less than 50%, potentially composed of soft atherosclerotic plaque with extensive thrombus.

**Image 3:**
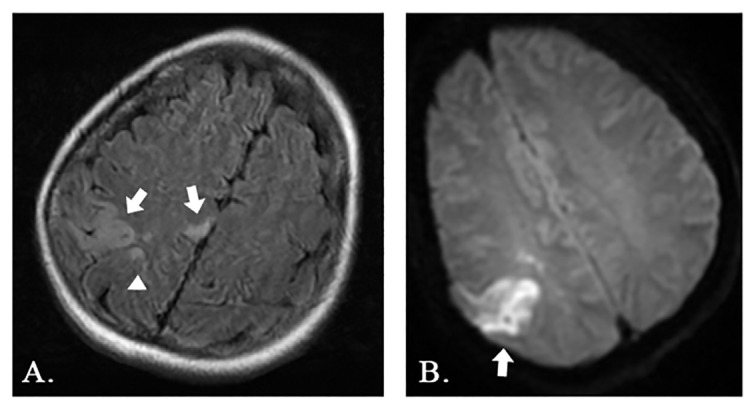
Brain magnetic resonance imaging showing (A) multiple foci of abnormal restricted diffusion predominantly involving the right parietal lobe (arrows) and right occipital lobe (arrowhead), and (B) hyperintense signal of the right parietal lobe on axial T2 fluid attenuated inversion recovery (FLAIR) sequence (arrow).

The diagnostic study was a CT angiography of the head and neck. As described by the radiologist, the patient sustained small, recent infarctions involving the right parietal lobe and potentially the right precentral gyrus. A soft atherosclerotic plaque with extensive thrombus was present in the right external carotid artery. The patient proceeded to have an MRI of the brain which showed acute infarcts in both the right anterior cerebral artery (ACA) and MCA distributions suggesting an embolic etiology, likely from the carotid plaque seen on the CT angiography, demonstrated in Images 2 and 3. An ultrasound of the carotid arteries identified a smooth plaque in the right proximal internal carotid artery. The patient was subsequently admitted to the neurology stroke service. Heparin was initiated for anticoagulation. Vascular surgery was consulted and the patient underwent a right carotid endarterectomy. After surgery, her left arm’s unusual movements had completely resolved. The sensory loss persisted, and she continued to feel the need to move, despite overall improvement in her symptoms. She was eventually transferred to acute rehabilitation, quit smoking, and was later discharged home.

## RESIDENT DISCUSSION (DR. POWELL)

In 1908, the German neurologist and psychiatrist Dr. Kurt Goldstein was bewildered by the strange behaviors manifested in one of his patients.^1^ This patient had reported that her left hand had a “will of its own,” and it had tried to choke her, forcing her to defend herself with her more obedient arm. Autopsy later identified infarctions in the right hemisphere and corpus callosum. It was not until 1972 that this constellation of symptoms was officially recognized as alien hand syndrome (AHS).^2^ It was coined by the French term “*le syndrome de la main étrangère*” (the sign of the foreign hand). Patients who present with AHS often do not recognize that the affected limb is a part of their own body. They state the limb feels foreign, complain of paresthesias, and are unable to voluntarily control its movement. This syndrome typically affects the hand but can also affect the leg.^3^ Initially, this syndrome was thought to be a post-surgical complication from corpus callosotomy or frontal lobe ablation.^4^ Today there are three main variants of AHS described in the literature.

Feinberg et al described two distinct syndromes: a frontal and a callosal variant.^5^ The frontal variant affects the lesions of the supplementary motor area, cingulate cortex, and medial frontal cortex, typically caused by a stroke in the anterior communicating artery territory with or without involvement of the corpus callosum.^6^ This variant commonly affects the dominant hand and presents with impulsive groping (where the hand seems to be constantly searching for nearby objects), compulsive manipulation of objects, and difficulty releasing objects.^7^ Associated conditions frequently encountered with this variant are those seen in frontal lobe lesions or frontal lobe ablations, including frontal release signs (grasp reflex, glabellar sign, palmomental reflex, etc), hemiparesis, and non-fluent aphasia.^8^

The callosal variant is typically caused by a callosal hemorrhage, infarct or callosotomy. Alien hand syndrome in this variant exclusively affects the non-dominant (left) hand in right-handed patients.^9^ The patient will present with intermanual conflict when the non-dominant hand is activated by conversation or voluntary activation of the dominant hand. Intermanual conflict is described as opposing, purposeful movements of the patient’s hands.^10^ The non-dominant hand behaves unilaterally, working against the dominant hand as if the hands are “fighting” against each other. There is minimal weakness associated with this variant. The associated findings include apraxia, tactile anomia, visual anomia, agraphia, and neglect.^11^

The final variant, and the type evidenced in this case presentation, is the posterior/opticosensory variant. The posterior variant is typically caused by a stroke in the parietal lobe or posterior cerebral artery territory, neurodegeneration of the parieto-occipital cortex (corticobasal syndrome) or Creutzfeldt-Jakob disease (CJD).^9^ As in our patient, this variant most commonly affects the non-dominant hand and is associated with parietal sensory deficits, including visual or sensory neglect, hemisomatognosia, body schema dysfunction, and spatial neglect.^12^ Patients exhibit strong feelings of estrangement from the affected limb and experience less complex motor activity such as limb levitation, ataxia, or non-purposeful or non-conflicting movements.^13^ These patients unintentionally withdraw the affected hand from the environment as an avoidance response or experience uncoordinated hand movements, which may be related to a task. In one reported case of corticobasal syndrome, exaggerated arm elevation only occurred while walking.^14^ The three different variants can present as a mixed picture depending on the lesion. For example, two CJD cases were reported with mixed anterior and posterior variant AHS. The patients had significant intermanual conflict and hemispatial neglect.^15^

Our patient sustained infarctions in both the ACA and MCA distributions. In a paper describing 100 patients suffering from ischemic strokes of the ACA (including subsequent infarctions in the corpus callosum, cingulate gyrus, or supplementary motor area), AHS occurred in 10% of medial frontal strokes.^16^ It is unknown how many MCA strokes involve AHS, due to the many additional symptoms presenting with MCA infarctions.

The ED workup includes basic labs, toxicological studies, ECG, and urine studies to look for alternative etiologies of the patient’s presentation. The first imaging study to obtain is CT of the head, as it will assess for etiologies such as space-occupying lesions and intracranial hemorrhage. Magnetic resonance imaging is the diagnostic imaging modality of choice as it will show both infarcted tissue, areas of edema, and salvageable brain tissue. As in our patient, angiography and ultrasound helped isolate and localize plaques and vessel stenosis. Patients with AHS should be admitted to the hospital for further evaluation, diagnostic studies, and management.

The treatment of AHS follows the initial management goals of an ischemic infarction. The underlying etiology may be treated with targeted blood pressure control, anticoagulation, specialist consultation, thrombolytics, embolectomy, and/or endarterectomy. There are specific techniques designed to target the uncontrolled “alien” limb after initial stabilization. Abnormal motor activity can be reduced by occupying the affected limb (holding a cane), placing the affected limb in a glove or mitt, or using progressive muscle relaxation (including botulism toxin or muscle relaxants). Mirror box therapy has been described as a way to conduct visual-spatial coaching. Mirror box therapy is hypothesized to improve AHS symptoms by restoring the relationship between motor intentions and visual feedback.^17^ The patient moves the unaffected limb in front of the mirror, hiding the “alien” limb from view. The reflection, where the affected limb should be, is perceived as their arm, and they can attribute sensorimotor features of that intact arm to their alien one.

In general, infarction-related AHS has a good prognosis. As in this case presentation, after standard-of-care stroke management, patient-tailored rehabilitation is very successful. The most debilitating aspects of AHS typically resolve within weeks to months with proper treatment and therapy. In a literature review, decreases in AHS symptoms occurred in 68% of patients, whereas symptoms persisted in 32%.^18^ Proper identification through an in-depth history and neurologic examination can aid in prompt diagnosis and treatment of this uncommon stroke variant.

## FINAL DIAGNOSIS

Alien hand syndrome secondary to acute non-dominant parietal lobe infarction.

## KEY TEACHING POINTS

- In addition to more prevalent stroke syndromes involving motor and sensory losses, consider stroke variants in patients who present with hyperkinetic movements and psychiatric overtones.
- Alien hand syndrome can present with abnormal hand movements and lack of subjective limb ownership.
- Alien hand syndrome can be caused by intracranial hemorrhage, infarction, or surgical alteration involving the frontal, callosal, and posterior brain territories.
- A thorough neurologic examination is critical to make the diagnosis of AHS, especially when faced with psychiatric bias.

## Figures and Tables

**Table 1:** Blood laboratory results of a 57-year-old female presenting with strange movements and shaking of her left arm.

Test Name	Patient Value	Reference Range
Complete blood count		
White blood cells	14.8 K/mcL	4.5 – 11 K/mcL
Hemoglobin	16.2 g/dL	11.9 – 15.7 g/dL
Hematocrit	47.6%	35.0 – 45.0%
Platelets	331 K/mcL	153 – 367 K/mcL
Differential		
Neutrophils	71.1%	42.6 – 74.5%
Lymphocytes	20.8%	20.8 – 50.5%
Monocytes	4.9%	2.0 – 10.3%
Eosinophils	2.0%	0.9 – 2.9%
Complete metabolic panel		
Sodium	143 mmol/L	136 – 145 mmol/L
Potassium	3.7 mmol/L	3.5 – 5.1 mmol/L
Chloride	111 mmol/L	98 – 107 mmol/L
Bicarbonate	28 mmol/L	21 –30 mmol/L
Glucose	116 mg/dL	70 – 99 mg/dL
Blood urea nitrogen	15 mg/dL	7 – 17 mg/dL
Creatinine	0.84 mg/dL	0.52 – 1.04 mg/dL
Calcium	9.4 mg/dL	8.6 – 10.2 mg/dL
Magnesium	2.2 mg/dL	1.6 – 2.6 mg/dL
Phosphorous	3.8 mg/dL	2.5 – 4.5 mg/dL
Total protein	7.7 g/dL	6.3 – 8.2 g/dL
Albumin	4.4 g/dL	3.2 – 4.6 g/dL
Total bilirubin	0.5 mg/dL	0.3 – 1.2 mg/dL
Aspartate aminotransferase	31 u/L	14 – 36 units/L
Alanine aminotransferase	19 u/L	0 – 34 units/L
Alkaline phosphatase	66 u/L	38 – 126 units/L
Additional Labs		
Troponin I	<0.02 ng/mL	<=0.06 ng/mL
Acetaminophen	<10.0 mcg/mL	<10.0 mcg/mL
Salicylate level	<1.0 mg/dL	<1.0 mg/dL
Ethanol level	<10.0 mg/dL	<10.0 mg/dL

*K*, thousands; *mcL*, microliter; *g*, grams; *dL*, deciliter; *mmol*, millimole; *L*, liter; *mg*, milligram; *u*, unit; *ng*, nanogram; *mcg*, microgram; *mL*, milliliter.

**Table 2:** Urine laboratory results of a 57-year-old female presenting with strange movements and shaking of her left arm.

Test Name	Patient Value	Reference Range
Urinalysis		
pH	6.0	5.0 – 8.0
Specific gravity	1.008	1.002 – 1.030
Glucose	Negative	Negative
Ketones	Negative	Negative
Nitrates	Negative	Negative
Leukocyte esterase	3+	Negative
White blood cells	26–50 count/uL	0 – 5/hpf
Red blood cells	26–50 count/uL	0 – 2/hpf
Squamous epithelial cells	6–10 count/uL	Negative
Bacteria	Negative	Negative
Urine Toxicology		
Amphetamine	Negative	Negative
Barbiturate	Negative	Negative
Benzodiazepine	Negative	Negative
Cannabinoid	Positive	Negative
Fentanyl	Negative	Negative
Methadone	Negative	Negative
Opiate	Positive	Negative
Oxycodone/oxymorphone	Negative	Negative
Phencyclidine	Negative	Negative
Additional Urine Studies		
Urine pregnancy	Negative	Negative

*μL*, microliter; *hpf*, high power field.
